# Development and validation of a serological marker-based nomogram for risk stratification of spinal brucellosis

**DOI:** 10.3389/fcimb.2026.1744062

**Published:** 2026-04-10

**Authors:** Jin-hua Yuan, Pan Du, Jia-qing Zhao, Zi-min Ma, Li-Na Ma, Xiang-Chun Ding

**Affiliations:** 1 The First Clinical Medical College of Ningxia Medical University, Yinchuan, Ningxia, China; 2 Ningxia Key Laboratory of Prevention and Control of Common Infectious Diseases, Yinchuan, China; 3 School of Basic Medicine, Ningxia Medical University, Yinchuan, China; 4 Weiluo Microbial Pathogens Monitoring Technology Co., Ltd. of Beijing, Beijing, China; 5 Department of Infectious Disease, General Hospital of Ningxia Medical University, Yinchuan, Ningxia, China; 6 Infectious Disease Clinical Research Center of Ningxia, Yinchuan, Ningxia, China

**Keywords:** brucellosis, nomogram, predictive model, restricted cubic splines (RCS), SHAP, spinal brucellosis

## Abstract

**Background:**

Spinal involvement is a major determinant of impaired quality of life in patients with brucellosis and may even be life-threatening. Efficient and cost-effective serological testing may enable earlier identification of spinal brucellosis.

**Objective:**

To develop and validate a nomogram based on routine serological indicators for individualized prediction of spinal brucellosis risk.

**Methods:**

We retrospectively collected clinical data from 427 patients with brucellosis admitted to the General Hospital of Ningxia Medical University between September 2021 and September 2025. Patients were classified into a non-spinal brucellosis group (n = 359) and a spinal brucellosis group (n = 68) according to the presence of spondylitis. Participants were randomly split into a training set and an internal validation set at a 7:3 ratio. After variable selection in the training set, independent predictors were identified using multivariable logistic regression and incorporated into a nomogram. Model performance was comprehensively assessed using the area under the receiver operating characteristic curve (AUC), calibration curves, the Brier score, and multiple classification metrics. Decision curve analysis (DCA) was used to evaluate clinical net benefit. SHAP was applied to interpret variable contributions, restricted cubic splines (RCS) were used to examine nonlinear relationships, and prespecified subgroup analyses were performed.

**Results:**

The final model included four pre-treatment serological markers, namely platelet count (PLT), platelet-to-lymphocyte ratio (PLR), interleukin-4 (IL-4), and Serum ferritin. The model showed moderate discrimination in the training and validation sets (AUC = 0.762, 95% CI: 0.692–0.831; and AUC = 0.664, 95% CI: 0.521–0.807, respectively), showing overall moderate calibration, with Brier scores of 0.121 and 0.125 in the training and validation cohorts, respectively. DCA indicated stable net benefit across reasonable threshold probabilities. SHAP analysis identified PLR as the strongest contributor to prediction. RCS analysis suggested linear associations for PLT, PLR, and serum ferritin, whereas IL-4 showed nonlinear relationships with risk. Subgroup analyses demonstrated generally consistent effect directions, but a significant interaction between IL-4 and diabetes status was observed.

**Conclusion:**

This nomogram enables individualized risk assessment for spondylitis among patients with brucellosis and may serve as a practical tool for preliminary screening and MRI decision-making in clinical practice.

## Introduction

Brucellosis is a highly contagious zoonosis caused by bacteria of the genus Brucella and remains widely distributed worldwide. It continues to threaten both animal and human health and imposes a substantial burden on health-care systems and socioeconomic development in endemic regions of many low- and middle-income countries, particularly in rural areas (El Ayoubi et al., 2024; Khairullah et al., 2024). Humans are mainly infected through direct contact with infected animal fluids, inhalation of contaminated aerosols, or ingestion via the gastrointestinal tract; a small proportion of cases are laboratory-acquired infections (Shakir, 2021).

The clinical presentation of brucellosis is highly heterogeneous. In the acute phase, patients commonly present with fever, profuse sweating, fatigue, and musculoskeletal pain. If infection persists, the disease may progress to a chronic course and lead to various disabling complications (Zheng et al., 2018a). Among these, spinal brucellosis is one of the most important causes of disability and mortality, with reported prevalence ranging from 2% to 60% (Rizkalla et al., 2021; Spernovasilis et al., 2024). Notably, therapeutic strategies differ substantially between uncomplicated brucellosis and spinal involvement. Uncomplicated disease is typically treated with a two-drug regimen (e.g., rifampicin plus doxycycline, RIF + DOX), whereas spinal brucellosis often requires triple therapy, with RIF and DOX administered for at least 12 weeks in combination with an aminoglycoside (El Ayoubi et al., 2024). Treatment duration and regimen selection largely depend on clinical manifestations and imaging assessment, and some patients ultimately require surgical intervention (El Ayoubi et al., 2024).

However, spinal brucellosis often has an insidious onset and lacks specific symptoms. In clinical practice, it is frequently misdiagnosed as lumbar muscle strain or tuberculous spondylitis, and delayed diagnosis and treatment are associated with a high risk of disability. Therefore, early identification of high-risk patients and timely diagnosis are of major clinical importance for preventing disease progression and improving outcomes. Although magnetic resonance imaging (MRI) can detect typical cases at an early stage, its high cost and limited availability in primary care settings preclude its use as a routine screening tool (Pu et al., 2024). Accordingly, an efficient, affordable, and accessible approach is urgently needed to identify brucellosis patients at high risk of spinal involvement and to optimize the use of imaging examinations.

Growing evidence highlights a pivotal role of CD4+T lymphocytes in the chronicity of brucellosis and the development of complications. T helper 1 (Th1) and Th2 cells are the earliest defined functional subsets of CD4+ T cells, and Th1 responses are essential for macrophage activation and clearance of intracellular Brucella (Qureshi et al., 2023; Wang et al., 2024). In chronic brucellosis, the pathogen can evade immune surveillance through multiple mechanisms, leading to CD4^+^ Th1-cell exhaustion and a skewing of T-cell differentiation toward a Th2 phenotype, thereby weakening pathogen clearance (Lu et al., 2025). Previous studies have explored the predictive value of C-reactive protein, various cytokines, and inflammation-related markers for brucellosis complications; however, most analyses focused on single indicators, and an integrated prediction model that combines multiple parameters and quantifies individual risk for spinal brucellosis remains lacking (Aktug Demir et al., 2014; Lin et al., 2020; Gao et al., 2022).

Given that serological parameters are readily available, low-cost, and biologically interpretable, we developed and validated, for the first time, a visual nomogram to enable early discrimination between uncomplicated brucellosis and spinal brucellosis. Model performance was comprehensively evaluated using the area under the receiver operating characteristic curve (AUC), calibration curves, the Brier score, and multiple classification metrics, and clinical net benefit was assessed by decision curve analysis (DCA). We anticipate that this tool will assist clinicians in selecting high-risk patients for early MRI examination, reduce missed and incorrect diagnoses, and ultimately improve outcomes and prognosis in patients with spinal brucellosis.

## Methods

### Study design and data source

This single-centre, retrospective cohort study consecutively enrolled 427 patients diagnosed with brucellosis at the General Hospital of Ningxia Medical University between 1 September 2021 and 1 September 2025. Standardised case report forms were used to collect all pre-treatment variables. The study protocol was approved by the Hospital’s Medical Ethics Committee (Approval No. KYLL-2025-0873, Yinchuan, China) and conducted in strict accordance with the principles of the Declaration of Helsinki. Written informed consent was obtained from all participants.

### Diagnostic criteria

Confirmed brucellosis: Patients were required to present compatible clinical manifestations plus one of the following: (1) detection of Brucella species in clinical specimens by nucleic acid amplification techniques, or (2) a standard tube agglutination test (SAT) titer of ≥1:160 in non-endemic areas or ≥1:320 in endemic areas (El Ayoubi et al., 2024).

Spinal brucellosis: clinical manifestations and serological evidence consistent with brucellosis, together with spinal MRI findings showing typical inflammatory changes, vertebral and/or intervertebral disc destruction, or abscess formation; in some patients, CT was additionally performed to evaluate the extent of bony destruction (Pu et al., 2024; Spernovasilis et al., 2024).

### Inclusion and exclusion criteria

Inclusion criteria: (1) Patients meeting the above diagnostic criteria; (2) Those who received at least one full course of standardised treatment and completed ≥3 months of follow-up.

Exclusion criteria: (1) Patients with incomplete clinical data or age ≤18 years; (2) Those with co-infections including tuberculosis, typhoid, paratyphoid, rheumatic fever, or other natural focal diseases, as well as individuals with immunodeficiency; (3) Patients with other infectious or immune-mediated spondylitis; (4) Long-term users of corticosteroids or immunosuppressants, and pregnant or lactating individuals.

### Variable definitions

Grouping Variable: Patients were stratified into two groups based on the presence or absence of brucellar spondylitis: uncomplicated brucellosis and spinal brucellosis. Covariates: These included demographic characteristics (sex, age, ethnicity, history of exposure, and occupation), comorbidities (hypertension, diabetes mellitus, and coronary heart disease), clinical manifestations (fever, fatigue, bone and joint pain, and hyperhidrosis), and treatment regimens. The regimens were categorized into: Rifampin + Doxycycline (RIF + DOX); Doxycycline + Rifampin + Fluoroquinolones (DOX + RIF + FQs); and Doxycycline + Rifampin + Cephalosporins (DOX + RIF + Cep). Predictor Variables: The analyzed serological parameters comprised haematological indices (white blood cell count [WBC], haemoglobin [HGB], platelet count [PLT], neutrophil count, lymphocyte count, neutrophil-to-lymphocyte ratio [NLR], and platelet-to-lymphocyte ratio [PLR]), coagulation and inflammatory markers (D-dimer, fibrinogen [FIB], lactate dehydrogenase [LDH], procalcitonin [PCT], high-sensitivity C-reactive protein [hs-CRP], C-reactive protein-to-albumin ratio [CAR], erythrocyte sedimentation rate [ESR], serum ferritin, interleukin-2 [IL-2], IL-4, interleukin-6 [IL-6], interleukin-10 [IL-10], interferon-gamma [IFN-γ], and tumour necrosis factor-alpha [TNF-α]), and immunological markers (CD4+/CD8+ T-cell ratio, serum immunoglobulin G [IgG], serum immunoglobulin A [IgA], serum immunoglobulin M [IgM], complement C3, and complement C4).

### Sample size consideration

Based on the events-per-variable (EPV) rule, at least 10 spinal brucellosis events were required per predictor in the final model. With four predictors and 68 spinal brucellosis events, the EPV criterion (≥10) was satisfied.

### Statistical analysis

All statistical analyses and figure generation were performed using R (version 4.4.3), and Adobe Illustrator was used for figure layout and font adjustment. Statistical methods were selected according to variable type and distribution. Categorical variables are presented as counts and percentages (%) and were compared using the chi-square test or Fisher’s exact test, as appropriate. Continuous variables were summarized as median and interquartile range [M (IQR)] and compared using the Mann−Whitney U test.

Participants were randomly split into a training set and an internal validation set (7:3), with stratification by spondylitis status to maintain a similar event proportion across cohorts. In the training set, variables associated with the outcome in univariable logistic regression (P < 0.05) were carried forward. After assessing multicollinearity, feature selection was performed using least absolute shrinkage and selection operator (LASSO) regression. Selected variables were entered into a multivariable logistic regression model to identify independent predictors and to construct the final model, which was visualized as a nomogram.

Model discrimination was quantified by the area under the receiver operating characteristic curve (AUC), and calibration was assessed using calibration curves. Overall performance was evaluated using the Brier score and additional classification metrics, and clinical utility was examined by decision curve analysis (DCA). Model interpretability was explored using SHapley Additive exPlanations (SHAP). Restricted cubic splines (RCS) were used to assess potential nonlinear associations for continuous predictors, and prespecified subgroup analyses were conducted. All tests were two-sided, with P < 0.05 considered statistically significant.

## Results

### Patient baseline characteristics

The baseline characteristics are shown in **Table 1**. A total of 427 brucellosis patients were included in this study, with 359 (84.07%) diagnosed with uncomplicated brucellosis and 68 (15.93%) with spinal brucellosis. No significant demographic differences were observed between the two groups. In terms of clinical manifestations, the incidence of bone and joint pain was significantly higher in the spinal brucellosis group compared to the uncomplicated brucellosis group (85.29% vs. 47.91%, P < 0.001), while the incidence of hyperhidrosis was significantly lower (26.47% vs. 44.01%, P = 0.010). No significant differences were found in other symptoms (e.g., fever, fatigue) (all P > 0.05).

**Table 1:** Baseline characteristics of 427 patients.

Characteristics	Overall (N = 427)	Uncomplicated brucellosis (N = 359)	Spinal brucellosis (N = 68)	P value
Demographic features				
Age (years), median (Q1, Q3)	52.00 (39.00, 60.00)	51.00 (38.00, 60.00)	54.00 (46.00, 59.00)	0.059
Sex, n (%)				0.823
Male	287 (67.21)	240 (66.85)	47 (69.12)	
Female	140 (32.79)	119 (33.15)	21 (30.88)	
Ethnic group, n (%)				>0.999
Han Chinese	283 (66.28)	238 (66.30)	45 (66.18)	
Ethnic minorities	144 (33.72)	121 (33.70)	23 (33.82)	
History of exposure, n (%)	230 (53.86)	189 (52.65)	41 (60.29)	0.304
Occupation, n (%)				0.607
Farmer	210 (49.18)	179 (49.86)	31 (45.59)	
Non-farmer	217 (50.82)	180 (50.14)	37 (54.41)	
Comorbidity, n (%)				
Hypertension	60 (14.05)	47 (13.09)	13 (19.12)	0.262
Diabetes	26 (6.09)	20 (5.57)	6 (8.82)	0.278
Coronary disease	26 (6.09)	20 (5.57)	6 (8.82)	>0.999
Clinical manifestations, n (%)				
Fever	317 (74.24)	272 (75.77)	45 (66.18)	0.132
Bone and joint pain	230 (53.86)	172 (47.91)	58 (85.29)	<0.001
Fatigue	340 (79.63)	283 (78.83)	57 (83.82)	0.439
Hyperhidrosis	176 (41.22)	158 (44.01)	18 (26.47)	0.010
Serological indicators, median (Q1, Q3)				
WBC(*109/L)	5.36 (4.01, 7.06)	5.30 (3.90, 6.87)	5.57 (4.29, 7.59)	0.086
HGB(g/L)	130.00 (116.00, 143.00)	131.00 (116.00, 143.00)	126.50 (114.50, 143.50)	0.692
PLT(g/L)	210.00 (145.00, 270.00)	199.00 (131.00, 262.00)	241.50 (187.00, 297.50)	<0.001
Neutrophil count(*109/L)	2.92 (2.00, 4.45)	2.80 (1.92, 4.26)	3.75 (2.46, 5.14)	0.006
Lymphocyte count(*109/L)	1.70 (1.22, 2.30)	1.72 (1.24, 2.30)	1.70 (1.10, 2.30)	0.445
NLR	1.82 (1.09, 2.76)	1.75 (1.07, 2.61)	2.16 (1.62, 3.59)	0.003
PLR	116.79 (75.44, 169.66)	112.54 (71.71, 160.32)	152.34 (106.60, 229.73)	<0.001
LDH(U/L)	239.00 (176.00, 382.00)	243.00 (179.00, 387.00)	197.50 (167.50, 362.50)	0.008
D-dimer(ug/ml FEU)	1.09 (0.63, 2.41)	1.17 (0.64, 2.58)	0.86 (0.62, 1.55)	0.036
FIB(g/L)	3.53 (2.68, 4.22)	3.41 (2.65, 4.17)	3.84 (3.08, 4.55)	0.015
PCT(ng/mL)	0.11 (0.05, 0.23)	0.12 (0.05, 0.24)	0.08 (0.05, 0.15)	0.141
hs-CRP(mg/L)	25.00 (7.80, 49.30)	26.80 (7.12, 49.70)	23.14 (10.39, 46.80)	0.803
CAR	0.73 (0.21, 1.52)	0.73 (0.19, 1.57)	0.71 (0.27, 1.29)	0.724
ESR(mm/h)	18.00 (7.00, 37.00)	18.00 (7.00, 38.00)	17.50 (8.50, 36.00)	0.764
Serum ferritin(ng/mL)	449.00 (224.00, 910.00)	472.00 (224.00, 952.00)	323.50 (214.00, 611.50)	0.009
IL-2(pg/mL)	0.26 (0.08, 3.05)	0.20 (0.08, 3.05)	0.75 (0.09, 4.22)	0.090
IL-4(pg/mL)	1.47 (0.06, 5.83)	0.75 (0.05, 4.67)	3.17 (0.63, 5.83)	<0.001
IL-6(pg/mL)	17.20 (7.13, 37.00)	17.20 (6.82, 36.20)	16.04 (10.66, 39.40)	0.403
IL-10(pg/mL)	4.80 (0.26, 11.05)	4.48 (0.26, 11.05)	5.71 (0.48, 12.47)	0.346
IFN-γ(pg/mL)	53.32 (12.63, 172.44)	53.32 (12.63, 172.44)	36.15 (12.63, 70.12)	0.508
TNF-α(pg/mL)	4.48 (0.32, 14.18)	4.17 (0.32, 14.18)	8.34 (1.60, 14.18)	0.123
CD4+T/CD8+T cell	1.67 (0.93, 1.98)	1.50 (0.85, 1.98)	1.82 (1.59, 2.39)	<0.001
Serum IgG protein(g/L)	11.90 (10.20, 13.60)	11.90 (10.20, 13.60)	11.85 (10.20, 12.60)	0.559
Serum IgA protein(g/L)	2.03 (1.61, 3.50)	2.03 (1.61, 3.55)	1.94 (1.61, 2.86)	0.134
Serum IgM protein(g/L)	1.14 (0.70, 2.48)	1.14 (0.70, 2.48)	1.15 (0.73, 2.48)	0.429
Complement C3(g/L)	1.13 (0.76, 1.57)	1.13 (0.76, 1.57)	1.10 (0.65, 1.57)	0.577
Complement C4(g/L)	0.49 (0.25, 0.61)	0.49 (0.24, 0.61)	0.49 (0.34, 0.61)	0.076

NLR, neutrophil-to-lymphocyte ratio; PLR, platelet-to-lymphocyte ratio; LDH, lactate dehydrogenase; FIB, fibrinogen; PCT, procalcitonin; hs-CRP, high-sensitivity C-reactive protein; CAR, C-reactive protein-to-albumin ratio; ESR, erythrocyte sedimentation rate; IFN-γ, interferon-gamma; TNF-α, tumor necrosis factor-alpha.

Serological comparisons showed higher levels of PLT, neutrophil count, NLR, PLR, FIB, IL-4, and CD4+T/CD8+ T cell ratio in the spinal brucellosis group (all P < 0.05), whereas LDH, D-dimer, and serum ferritin were significantly lower (all P < 0.05). No significant differences were observed in other markers (e.g., WBC, HGB, lymphocyte count, PCT, hs-CRP, CAR, ESR, IL-2/IL-6/IL-10, IFN-γ, TNF-α, IgG/IgA/IgM, complement C3/C4, etc.) (all P > 0.05).

### Feature selection

**Figure 1:**
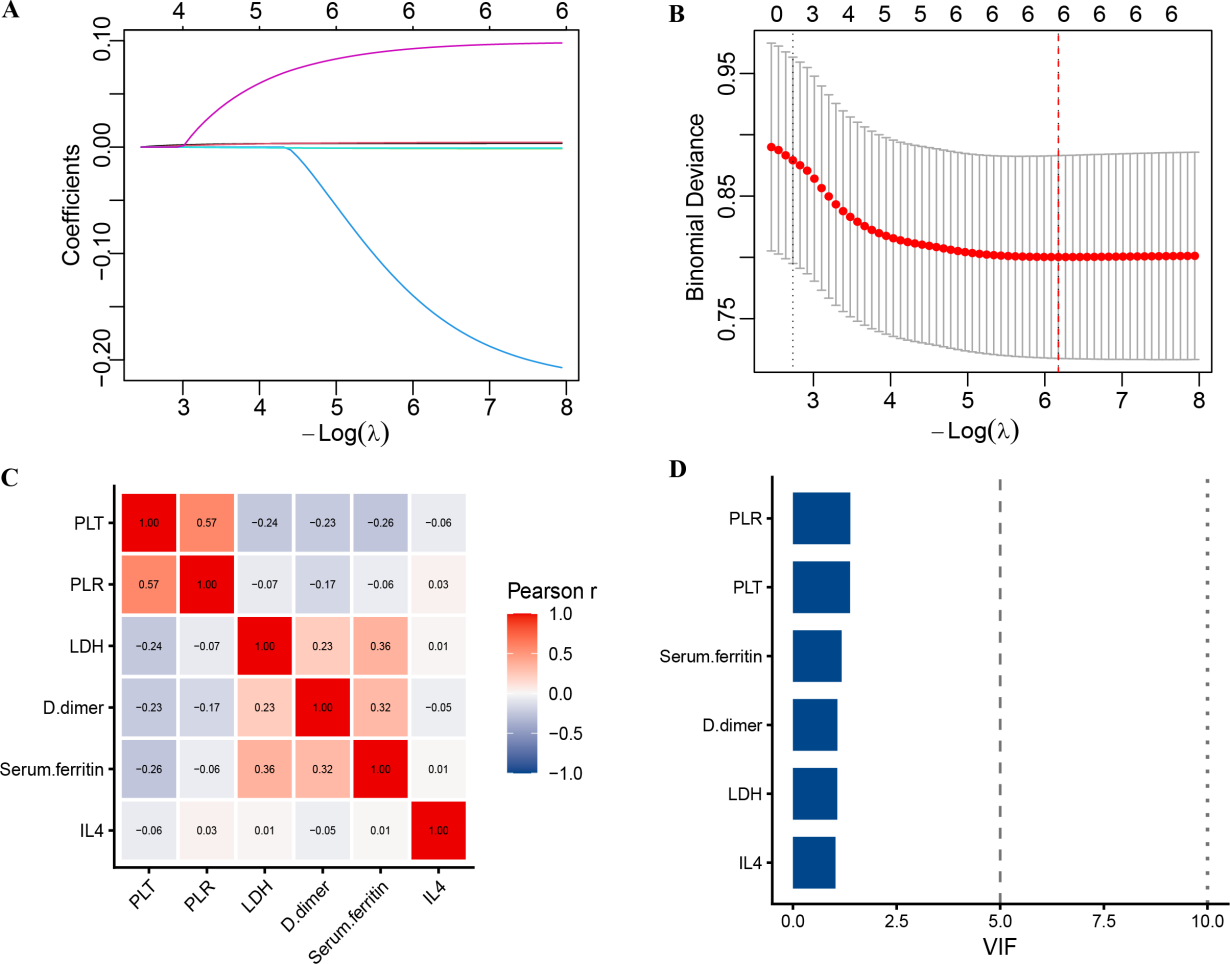
**(A)** Coefficient path plot of the LASSO regression. **(B)** Cross-validation plot of the LASSO regression. **(C)** Correlation heatmap for assessing multicollinearity. **(D)** Bar chart of variance inflation factors (VIFs).

To select potential predictors, univariable logistic regression was performed on all serological parameters in the training cohort. Univariable analysis identified PLT, PLR, LDH, D-dimer, serum ferritin, and IL-4 as significantly associated with spinal brucellosis (all P < 0.05; see **Table 2**). LASSO regression was then used for feature reduction, and the same six variables were retained based on the minimum lambda criterion (lambda.min = 0.002) for further analysis (see Table **2** and **Figures 1A**, **B**). Correlation analysis and variance inflation factor (VIF) testing revealed no significant collinearity among these variables, with all VIF values < 5 (see **Figures 1C**, **D**).

**Table 2:** All serological parameters were analyzed using univariate logistic regression.

Variables	β	S.E	Z	P	OR (95%CI)
WBC(*109/L)	0.04	0.05	0.77	0.444	1.04 (0.95 ~ 1.13)
HGB(g/L)	0.01	0.01	1.08	0.279	1.01 (0.99 ~ 1.02)
**PLT(g/L)**	0.01	0.00	3.92	<.001	1.01 (1.01 ~ 1.01)
Neutrophil count(*109/L)	-0.01	0.03	-0.30	0.762	0.99 (0.94 ~ 1.05)
Lymphocyte count(*109/L)	-0.20	0.19	-1.08	0.282	0.82 (0.56 ~ 1.18)
NLR	-0.01	0.04	-0.25	0.801	0.99 (0.92 ~ 1.07)
**PLR**	0.01	0.00	3.42	<.001	1.01 (1.01 ~ 1.01)
**LDH(U/L)**	-0.01	0.00	-2.29	0.022	0.99 (0.99 ~ 0.99)
**D-dimer(ug/ml FEU)**	-0.32	0.14	-2.35	0.019	0.72 (0.55 ~ 0.95)
FIB(g/L)	-0.00	0.06	-0.04	0.969	1.00 (0.89 ~ 1.12)
PCT(ng/mL)	-1.32	0.88	-1.51	0.132	0.27 (0.05 ~ 1.49)
hs-CRP(mg/L)	-0.00	0.01	-0.75	0.451	1.00 (0.99 ~ 1.01)
CAR	-0.17	0.18	-0.92	0.360	0.84 (0.59 ~ 1.21)
ESR(mm/h)	-0.00	0.00	-0.33	0.739	1.00 (0.99 ~ 1.01)
**Serum ferritin(ng/mL)**	-0.01	0.00	-2.87	0.004	0.99 (0.99 ~ 0.99)
IL-2(pg/mL)	0.03	0.07	0.43	0.664	1.03 (0.90 ~ 1.19)
**IL-4(pg/mL)**	0.08	0.04	2.25	0.024	1.09 (1.01 ~ 1.17)
IL-6(pg/mL)	-0.00	0.00	-0.12	0.905	1.00 (0.99 ~ 1.01)
IL-10(pg/mL)	0.02	0.02	1.08	0.280	1.02 (0.98 ~ 1.07)
IFN-γ(pg/mL)	0.00	0.00	0.83	0.404	1.00 (1.00 ~ 1.00)
TNF-α(pg/mL)	0.00	0.02	0.29	0.775	1.00 (0.97 ~ 1.03)
CD4+T/CD8+T cell	0.15	0.08	1.75	0.079	1.16 (0.98 ~ 1.37)
Serum IgG protein(g/L)	-0.01	0.01	-0.81	0.417	0.99 (0.98 ~ 1.01)
Serum IgA protein(g/L)	-0.11	0.09	-1.20	0.230	0.89 (0.74 ~ 1.07)
Serum IgM protein(g/L)	0.01	0.11	0.08	0.937	1.01 (0.81 ~ 1.26)
Complement C3(g/L)	0.02	0.32	0.08	0.940	1.02 (0.54 ~ 1.93)
Complement C4(g/L)	0.90	0.52	1.75	0.080	2.46 (0.90 ~ 6.76)

Variables in bold were selected by LASSO. OR, odds ratio; CI, confidence interval.

### Multivariable logistic regression analysis

The six features selected by LASSO were included in multivariable logistic regression models, adjusting for potential confounders. Model 1 adjusted for age, sex, and ethnicity; Model 2 further adjusted for history of exposure, occupation, hypertension, diabetes, and coronary disease; and Model 3 additionally adjusted for fever, bone and joint pain, fatigue, hyperhidrosis, and therapeutic regimen. In Model 3, PLT (OR = 1.952, 95% CI: 1.299–2.933, p = 0.001), PLR (OR = 1.562, 95% CI: 1.121–2.178, p = 0.009), and IL-4 (OR = 1.765, 95% CI: 1.207–2.579, p = 0.003) were identified as independent positive predictors of spinal brucellosis, whereas LDH (OR = 0.549, 95% CI: 0.321–0.941, p = 0.029), D-dimer (OR = 0.098, 95% CI: 0.011–0.872, p = 0.037), and serum ferritin (OR = 0.452, 95% CI: 0.260–0.785, p = 0.005) were identified as independent negative predictors (see **Table 3**). Considering clinical applicability and following consultation with specialist clinicians, variables with p < 0.01 were selected for subsequent analysis.

**Table 3:** Multivariable logistic regression.

Predictor	Model 1		Model 2		Model 3	
	OR (95%CI)	p	OR (95%CI)	p	OR (95%CI)	p
PLT	2.000 (1.423, 2.811)	<0.001	1.943 (1.377, 2.742)	<0.001	1.952 (1.299, 2.933)	0.001
PLR	1.630 (1.221, 2.177)	<0.001	1.707 (1.252, 2.327)	<0.001	1.562 (1.121, 2.178)	0.009
LDH	0.580 (0.372, 0.904)	0.016	0.527 (0.321, 0.867)	0.012	0.549 (0.321, 0.941)	0.029
D-dimer	0.108 (0.017, 0.690)	0.019	0.089 (0.013, 0.620)	0.015	0.098 (0.011, 0.872)	0.037
Serum.ferritin	0.488 (0.310, 0.768)	0.002	0.407 (0.248, 0.666)	<0.001	0.452 (0.260, 0.785)	0.005
IL4	1.340 (1.011, 1.776)	0.042	1.389 (1.014, 1.903)	0.041	1.765 (1.207, 2.579)	0.003

Model 1: Adjusted for age, sex, and ethnic group. Model 2: Further adjusted for history of exposure, occupation, hypertension, diabetes, and coronary disease. Model 3: Additionally adjusted for fever, bone and joint pain, fatigue, hyperhidrosis, and therapeutic regimen.

### Prediction model construction and evaluation

**Figure 2:**
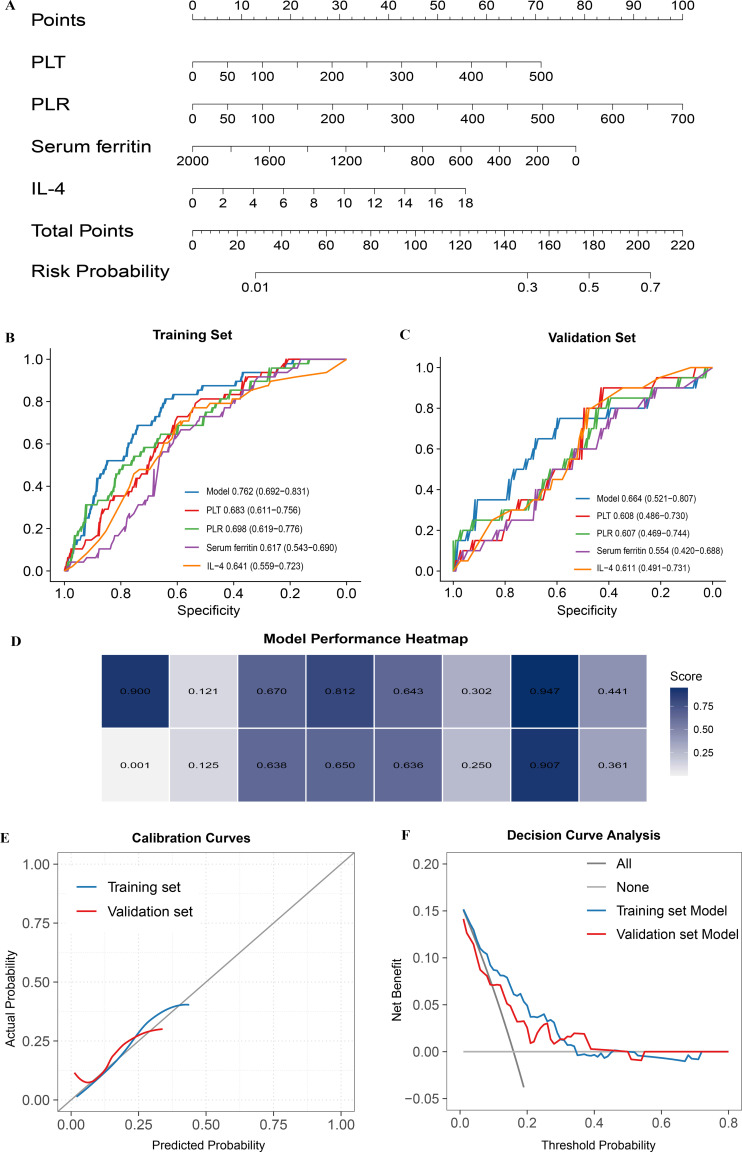
Development and validation of the nomogram prediction model. **(A)** Nomogram constructed using PLT, PLR, IL-4, and serum ferritin. **(B)** ROC curves of individual predictors and the nomogram in the training cohort. **(C)** ROC curves of individual predictors and the nomogram in the validation cohort. **(D)** Model Performance Heatmap. **(E)** Calibration curves of the nomogram in the training and validation cohorts. **(F)** Decision curve analysis (DCA) of the nomogram in the training and validation cohorts.

Based on four selected predictors (PLT, PLR, serum ferritin, and IL-4), a prediction model was developed and presented as a nomogram (**Figure 2A**). In the training cohort, the model achieved an AUC of 0.762 (95% CI: 0.692–0.831), demonstrating better discriminative performance than any individual marker (F**igure 2B**). In the validation cohort, the AUC was 0.664 (95% CI: 0.521–0.807), and the model still generally outperformed PLT, PLR, serum ferritin, and IL-4 used alone, indicating acceptable discrimination (**Figure 2C**). Calibration curves showed good agreement between predicted and observed probabilities in the training and validation cohort (**Figure 2E**). Consistently, the Hosmer-Lemeshow (HL) test yielded a P value of 0.900 in the training cohort, indicating good calibration, whereas the corresponding P value in the validation cohort was 0.001, suggesting moderate calibration performance overall. Comprehensive performance metrics were as follows: in the training set, the Brier score was 0.121, accuracy was 0.670, sensitivity was 0.812, specificity was 0.643, PPV was 0.302, NPV was 0.947, and the F1 score was 0.441; in the validation set, the corresponding values were 0.125, 0.638, 0.650, 0.636, 0.250, 0.907, and 0.361, respectively (**Figure 2D**). Decision curve analysis (DCA) further indicated that, within a threshold probability range of 10% to 40%, using the model to guide MRI screening resulted in higher clinical net benefit compared to “all test” or “no test” strategies.

### SHAP analysis

**Figure 3:**
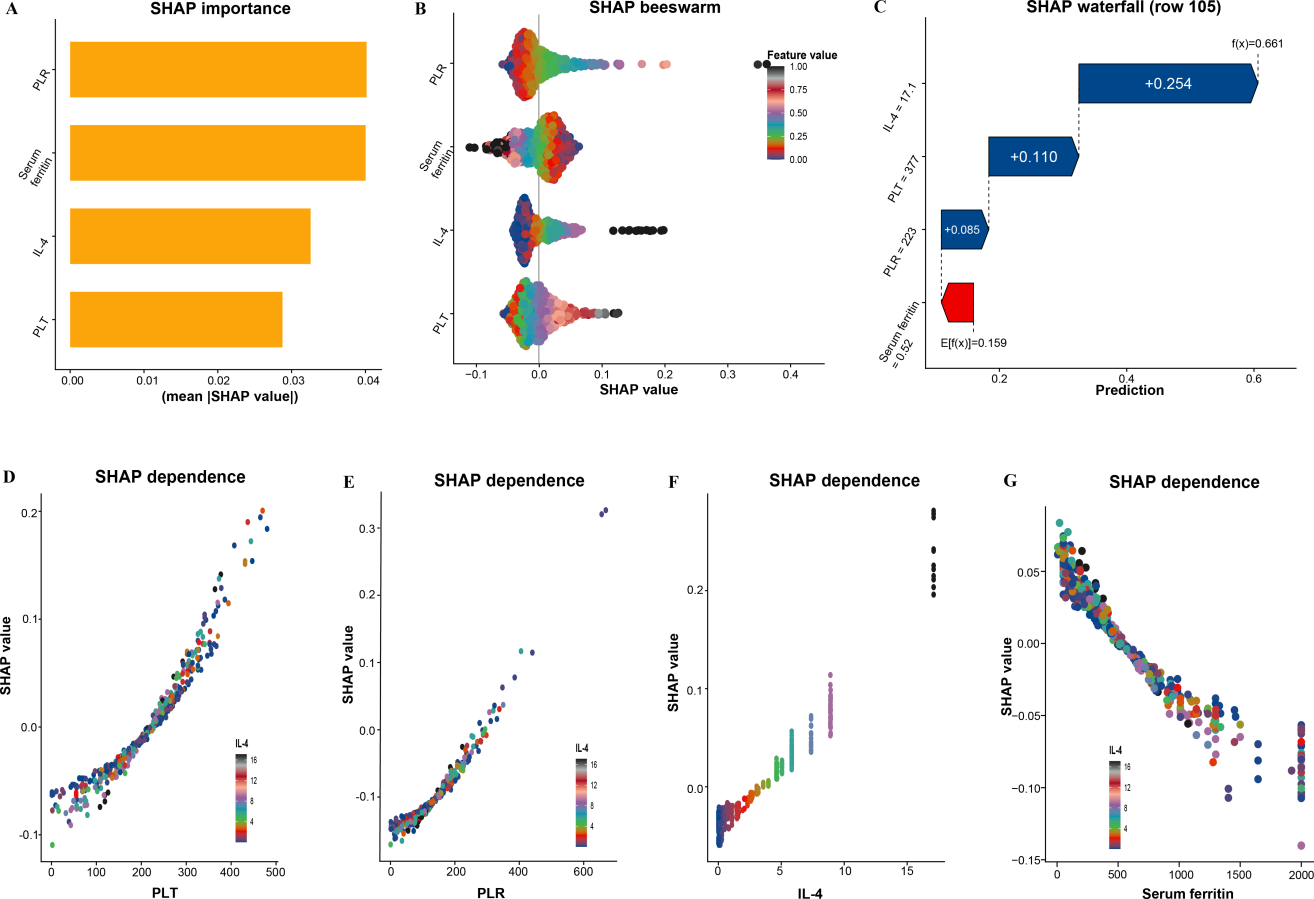
SHAP interpretation of model for predicting spinal brucellosis. **(A)** Global feature importance ranked by mean absolute SHAP value (mean |SHAP|). **(B)** SHAP beeswarm summary plot showing the distribution of SHAP values for each predictor across all individuals; points are colored by the corresponding feature value (low to high). **(C)** SHAP waterfall plot for a representative individual (row 105), illustrating how each predictor shifts the model output from the baseline expected value to the final predicted probability. **(D-G)** SHAP dependence plots for PLT, PLR, IL-4, and serum ferritin.

To enhance model interpretability, SHapley Additive exPlanations (SHAP) were used to quantify and visualize the contribution of each variable. Global importance analysis revealed that PLR had the highest contribution (**Figure 3A**). A summary plot illustrated the relationship between variable values and SHAP values, showing that higher values of IL-4, PLT, and PLR were associated with positive SHAP values, increasing the probability of disease. In contrast, higher serum ferritin levels were generally associated with lower SHAP values, suggesting a negative contribution to disease risk (**Figure 3B**). In the waterfall plot for a representative individual, the baseline output was E[f(x)] = 0.159, which increased to f(x) = 0.661 after incorporating the individual’s characteristics (**Figure 3C**). Among these variables, IL-4 contributed most to the increase in predicted risk, whereas serum ferritin showed a certain offsetting effect. Dependency plots showed a clear dose–response trend between variables and SHAP values (**Figures 3D–G**). PLT and PLR continuously increased the probability of disease, while IL-4 demonstrated a stepwise increase in SHAP values, suggesting a potential nonlinear amplification effect. In contrast, serum ferritin showed an overall negative association with SHAP values.

### Nonlinear testing and subgroup analysis

**Figure 4:**
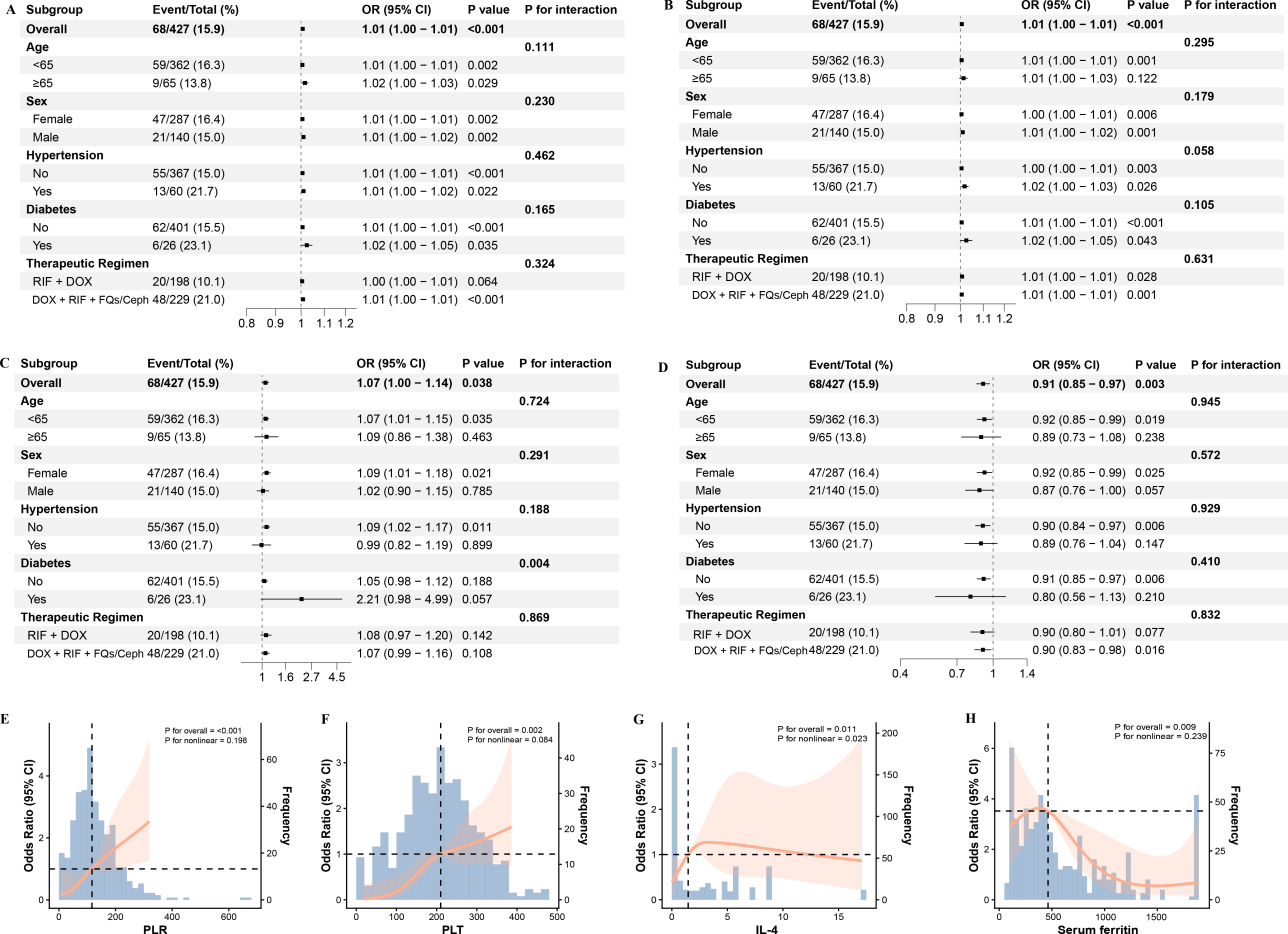
Subgroup effects and restricted cubic spline (RCS) analyses of key variables in the model. **(A–D)** Associations of PLT, PLR, IL-4, and serum ferritin across prespecified subgroups (age, sex, hypertension, diabetes, and treatment regimen). **(E–H)** RCS dose–response relationships between PLR, PLT, IL-4, and serum ferritin and the outcome; the orange curve indicates the adjusted odds ratio (OR).

In prespecified subgroup analyses (age, sex, comorbidities, treatment regimen), PLT, PLR, and IL-4 were consistently associated with an increased risk of spinal brucellosis, whereas serum ferritin was associated with a decreased risk of spinal brucellosis (**Figures 4A–D**). No significant interaction effects were observed in most comparisons. (P for interaction > 0.05). However, IL-4 exhibited significant variation across diabetes subgroups (P for interaction = 0.004), suggesting potential heterogeneity in its association with the outcome between diabetic and non-diabetic populations. RCS analysis indicated statistically significant overall associations between PLR, PLT, IL-4, and serum ferritin and the risk of spinal brucellosis (P for overall < 0.05) (**Figures 4E-H**). Nonlinear tests revealed linear relationships for PLR, PLT, and serum ferritin (P for nonlinear > 0.05) and nonlinear relationships for IL-4 (P for nonlinear < 0.05).

## Discussion

In this retrospective cohort study, we developed and internally validated a nomogram based on four serological parameters (PLT, PLR, IL-4, and serum ferritin) to individually estimate the risk of spinal involvement in patients with brucellosis. The model demonstrated moderate discrimination, moderate calibration, and tangible clinical utility in the internal validation cohort. Spinal brucellosis is among the most common complications of brucellosis and represents a major cause of chronic pain and disability (Wu et al., 2025). In endemic areas, early diagnosis of spinal brucellosis is critical to improving outcomes. Our model addresses this clinical need by providing an easy-to-use and cost-effective quantitative tool that may facilitate initial risk stratification and imaging decision-making, particularly in settings where access to advanced imaging is limited.

SHAP analyses identified PLR as the most influential predictor, followed by serum ferritin, IL-4, and PLT, indicating that systemic inflammatory burden and iron-related host responses may both play important roles in risk stratification for spinal involvement. The role of T lymphocytes in brucellosis has gained increasing attention in recent years. As an intracellular pathogen, Brucella is primarily controlled by cell-mediated immunity (Zheng et al., 2018b). Activated CD8+ T cells and CD4+ T helper 1 (Th1) responses are essential for bacterial clearance (Baldwin and Goenka, 2006; Durward et al., 2012; Durward-Diioia et al., 2015). partly through the production of cytokines such as interferon-γand IL-2, which reinforce Th1 function and enhance macrophage bactericidal activity (Skendros and Boura, 2013). Th1 and T helper 2 (Th2) cells represent the earliest defined functional subsets of CD4+ T cells, and increased Th2 responses can suppress Th1-mediated immunity; this balance is crucial for controlling the initiation and progression of Brucella infection (Zheng et al., 2018b). Prior evidence suggests that Th1 activation contributes to protective immunity, whereas Th2 responses may exacerbate disease (Rafiei et al., 2006). As a canonical Th2 cytokine, elevated IL-4 is closely linked to a shift from protective Th1 responses toward a potentially detrimental Th2 profile, which may impede pathogen clearance and promote chronic inflammatory foci. In our study, higher IL-4 levels in patients with spinal brucellosis are consistent with the proposed contribution of Th1/Th2 imbalance to chronic brucellosis. Nevertheless, IL-4 represents only one component of the broader Th2 cytokine network; elevation of a single cytokine is insufficient to fully characterize immune polarization. Here, IL-4 may be better interpreted as a surrogate marker reflecting a wider immunologic shift toward Th2 dominance. Such a change in the immune microenvironment could plausibly weaken clearance of intracellular Brucella, thereby facilitating bacterial persistence and local colonization in spinal tissues.

We also found that PLT and PLR were independent risk factors, and restricted cubic spline analyses suggested linear associations with spinal brucellosis risk, indicating relatively stable incremental effects across their ranges. PLR, a widely used systemic inflammation index, has shown value for prognosis assessment across diseases involving multiple organ systems (Zhou et al., 2014; Balta and Ozturk, 2015; Rajalingam et al., 2022). Elevated PLR typically reflects two processes: increased platelet count and a relative reduction in lymphocyte count. Platelets can amplify local inflammation by releasing diverse inflammatory mediators, whereas lymphopenia may indicate immune exhaustion or suppressed cellular immunity during persistent intracellular infection. Our findings align with prior reports showing higher PLR levels in complicated brucellosis (Sen et al., 2019). Together, these results support the concept that spinal brucellosis arises from the combined effects of inflammation and immune dysregulation. Notably, lymphopenia has been observed in blood samples from brucellosis patients since early investigations (Crosby et al., 1984), suggesting that immune fatigue may occur more readily during sustained infection. In addition, serum ferritin was retained in the final model as an independent negative predictor, and both SHAP and RCS analyses consistently suggested an overall inverse and predominantly linear association with spinal brucellosis risk. Serum ferritin is both a marker of iron storage and an acute-phase reactant, and its levels are shaped by inflammation, iron redistribution, and persistent infection ((Kell et al., 2014; Wang et al., 2010; Garcia-Casal et al., 2021). In brucellosis, lower serum ferritin may therefore reflect a distinct host-response pattern associated with spinal involvement, rather than simply milder inflammation, possibly involving chronic consumption, iron dysregulation, or immunometabolic remodeling (Arica et al., 2012). However, this association should be interpreted cautiously, as ferritin is influenced by multiple factors and does not necessarily indicate causality.

Compared with previous studies that largely focused on age, diagnostic delay, or single inflammatory/cytokine markers, our model integrates multidimensional inflammatory and immunological information to provide a more comprehensive, quantitative assessment of spinal involvement risk (Gao et al., 2022). This study has several strengths. First, it targets spinal involvement—one of the most clinically challenging and frequently delayed complications—and provides a practical tool for early risk stratification aligned with a clear clinical use case. Second, the predictors are mainly derived from routine hematologic and immunologic tests, making the model feasible and potentially scalable in resource-limited settings. Third, we used stratified random sampling by spinal involvement to create training and validation cohorts and systematically evaluated model performance across discrimination, calibration, the Brier score and other classification metrics, and decision curve analysis. Finally, we incorporated SHAP and restricted cubic splines to improve interpretability and to identify potential nonlinear associations, thereby bringing the model closer to biologically plausible processes.

## Limitations

Several limitations should be acknowledged. First, as a single-center retrospective study, selection bias cannot be fully excluded. Differences in circulating strain profiles, host genetic background, and comorbidity patterns across regions may affect generalizability; therefore, external validation in multicenter populations and across different levels of care is needed. Second, validation was internal only, and the relatively small number of spinal brucellosis events in the validation cohort likely contributed to the wide confidence interval of the AUC, introducing uncertainty in performance estimates. Without adequate external validation, using the model to guide MRI referral may lead to unnecessary imaging in some settings or increase missed diagnoses in others. Third, although we adjusted for multiple clinical confounders, residual confounding from unmeasured factors remains possible, including host genetic polymorphisms, strain virulence, prior antibiotic exposure, and more granular measures of animal-contact intensity and duration. Incorporating these factors in future studies may further improve model robustness and predictive accuracy. Accordingly, the most urgent next step is to conduct multicenter prospective cohort studies in diverse epidemiological contexts to externally validate and, if necessary, recalibrate the nomogram, and to assess its real-world impact on MRI utilization, diagnostic timeliness, and clinical outcomes. Following external validation, implementation strategies should be explored to support standardized use in outpatient care, particularly in primary health-care settings.

## Conclusion

In summary, we developed and internally validated a serology-based nomogram that enables individualized quantification of spinal involvement risk in brucellosis patients and demonstrated stable performance in terms of discrimination, calibration, and decision-analytic benefit. With further confirmation in multicenter prospective studies, this model may become a useful adjunct to optimize MRI use, promote earlier diagnosis and treatment, and ultimately reduce the burden of spinal brucellosis.

## Data Availability

The raw data supporting the conclusions of this article will be made available by the authors, without undue reservation.
